# The Association between Loneliness, Mental Well-Being, and Self-Esteem among Adolescents in Four Nordic Countries

**DOI:** 10.3390/ijerph18147405

**Published:** 2021-07-11

**Authors:** Nelli Lyyra, Einar Baldvin Thorsteinsson, Charli Eriksson, Katrine Rich Madsen, Asko Tolvanen, Petra Löfstedt, Raili Välimaa

**Affiliations:** 1 Faculty of Sport and Health Sciences, University of Jyväskylä, FI-40014 Jyväskylä, Finland; raili.valimaa@jyu.fi; 2 Faculty of Medicine and Health, University of New England, Armidale, NSW 2351, Australia; ethorste@une.edu.au; 3 Department of Public Health Sciences, Stockholm University, SE-10691 Stockholm, Sweden; charli.eriksson@su.se; 4 The National Institute of Public Health, University of Southern Denmark, DK-5230 Odense, Denmark; krma@sdu.dk; 5 Faculty of Education and Psychology, University of Jyväskylä, FI-40014 Jyväskylä, Finland; asko.tolvanen@jyu.fi; 6 Department of Public Health and Community Medicine, Sahlgrenska Academy, University of Gothenburg, 40530 Gothenburg, Sweden; petra.lofstedt@gu.se

**Keywords:** loneliness, positive mental health, mental well-being, self-esteem, adolescents, Nordic countries, public health

## Abstract

Positive mental health is central to adolescent well-being. The present study examines the prevalence of loneliness and positive mental health indicators (mental well-being and self-esteem) in four Nordic countries and associations between loneliness, mental well-being, and high self-esteem. This study is based on data from the Health Behaviour in School-aged Children (HBSC) study which was conducted in 2018 in Denmark, Finland, Iceland, and Sweden. Participants were 5883 15-year-old boys and girls. To examine the associations between loneliness, mental well-being, and self-esteem, structural equation modeling (SEM) was applied. In the comparison of Nordic countries, the prevalence of loneliness was highest among Finnish and Icelandic adolescents. High mental well-being and high self-esteem were most prevalent in Denmark and Sweden. In general, boys scored higher on positive mental health indicators and girls on loneliness. Loneliness was also a strong indicator of low mental well-being and low self-esteem in all Nordic countries. Loneliness is not only associated with mental health problems such as anxiety and depression, but it is also a risk factor for adolescents’ positive mental health. Positive mental health is important for healthy maturation and there is a need to develop initiatives to reduce adolescent loneliness and so support positive development.

## 1. Introduction

Loneliness and mental health issues are common and interrelated problems in adolescence [1]. Adolescence is a period in life characterized by heightened sensitivity to social stimuli and an increased need for peer interaction [2]. In addition, in adolescence individualization, identity exploration, and cognitive and physical maturation increase the risk of perceived social isolation and feelings of loneliness [3].

Loneliness is a negative feeling caused by a discrepancy between desired and actual interpersonal relationships [4]. Loneliness, especially when experienced often or over a prolonged period, is associated with various physical and psychological health problems and risk behaviors [5,6,7]. Among adolescents, loneliness is associated with, for example, poorer self-rated health and sleep problems [8], psychosomatic symptoms [9], anxiety, and depression [10,11,12,13]. Loneliness is also linked to various physical disorders such as diabetes and coronary heart disease [14]. A longitudinal study of 8 to 11-year-old children showed a high level of loneliness in childhood was associated with higher levels of depressive symptoms, poorer general health, and a higher prevalence of sleep disturbance at pre-adolescence [15]. Similarly, a longitudinal study with data collected at 2-year intervals from participants aged 7 to 17 years, showed that prolonged loneliness in childhood and adolescence was associated with depressive symptoms, a higher frequency of visits to the doctor, and lower perceived general health at age 17 [1].

The World Health Organization has declared positive mental health to be the “foundation for wellbeing and effective functioning for both the individual and the community” and has defined it as “a state of well-being in which the individual realizes his or her own abilities, can cope with the normal stresses of life, can work productively, and is able to make a contribution to his or her community” [16] (p. 12). Mental health is more than the absence of mental health problems and disorders. It is a concept that includes dimensions of hedonic (positive feelings, affect, emotions) and eudemonic (positive functioning, mindset, and relationships) well-being [17,18,19]. Adolescence is an important period for positive mental health development [20] and is affected positively by social support from multiple sources [21]. Although most adolescents report high levels of mental well-being [22], there is an increasing number of adolescents reporting feeling sad and hopeless [23].

Self-esteem is an important part of an individual’s self-concept and is regarded as essential for positive mental health and functioning during adolescence and later in life [24]. Rosenberg [25] defines self-esteem as the individual’s set of thoughts and feelings about his or her own worth and importance, which reflects the notion of “global” self-esteem or self-worth. Self-esteem uses the evaluative and affective dimensions of self-concept and is susceptible to multiple internal and external influences and changes during adolescence [26].

Experiencing loneliness, low mental well-being, and low self-esteem are common and interrelated problems in adolescence. Low self-esteem relates to concurrent and subsequent feelings of loneliness, and self-esteem and loneliness reciprocally influence one another [27]. Previous research indicates that there is a strong association between loneliness and adverse mental health [28]. However, there is a need to increase understanding of how loneliness is associated with positive mental health indicators.

The present study employed data from 15-year-old adolescents in four Nordic countries: Denmark, Finland, Iceland, and Sweden. The aims were to (a) compare the prevalence of loneliness, mental well-being, and high self-esteem across the four Nordic countries; (b) examine how loneliness is associated with mental well-being and self-esteem among Nordic adolescents, and (c) examine how strongly loneliness explains the variation of mental wellbeing and self-esteem in Sweden, Finland, Denmark, and Iceland. The Nordic countries are considered to be quite homogenous in terms of social and cultural heritage, and they represent a group of relatively comparable welfare states [29].

## 2. Materials and Methods

### 2.1. Data Collection

Data were collected from among Nordic adolescents from Denmark, Finland, Iceland, and Sweden as part of the Health Behaviour in School-aged Children (HBSC) study in 2018. The HBSC study is an international World Health Organization collaborative study that uses cross-sectional surveys carried out every four years among 11-, 13-, and 15-year-old students [30]. Nationally representative data sets are ensured using random cluster sampling [31].

The dataset for the present study was formed based on HBSC Nordic data and those Nordic countries that included an item on loneliness in the data collection round in 2017/2018. All countries in the HBSC study complied with relevant ethical and data gathering standards. Students answered the questionnaire voluntarily and anonymously during school hours by paper and pen (Sweden) or electronically (Finland, Denmark, and Iceland).

### 2.2. Participants

Participants were 5883 adolescents from 9th or 10th grade, depending on which grade level corresponds to age 15 years in each participating country. A similar percentage of boys and girls participated in each country (Denmark: total 936, boys 51%; Finland total 1146, boys 50%; Iceland total 2195, boys 49%; Sweden total 1606, boys 48%) and no statistical difference was observed for gender distribution within countries (χ²(3) = 2.48, *p* = 0.480).

### 2.3. Measures

Loneliness was assessed using a single item on global loneliness. In all countries other than Sweden, loneliness was assessed by the question ‘Do you feel lonely?’ with four response categories: 1 (Yes, very often), 2 (Yes, quite often), 3 (Sometimes), and 4 (No). In Sweden, adolescents were asked ‘How often do you feel lonely?’ with five response categories: 1 (Always), 2 (Most of the time), 3 (Sometimes), 4 (Rarely), and 5 (Never). For Swedish data the response categories 4 and 5 were combined to match the response categories used in other Nordic countries. To analyze the prevalence of frequent loneliness, the four response categories were dichotomized representing lonely (“yes, very often”/“yes, often”) and not lonely (“yes, sometimes”/“no”). Loneliness was used as a continuous variable in structural equation modeling in which it was set as a predictor variable. The direct, single-item measure of loneliness is highly correlated with the multi-item UCLA Loneliness Scale, which is an indirect measure of loneliness [8,32].

Mental well-being was assessed using the short version of the Warwick-Edinburgh Mental Well-being Scale (SWEMWBS), which is a valid measure of positive mental well-being for adolescents [33,34,35,36]. The short scale consists of seven items: (1) ‘I’ve been feeling optimistic about the future’, (2) ‘I’ve been feeling useful’, (3) ‘I’ve been feeling relaxed’, (4) ‘I’ve been dealing with problems well’, (5) ‘I’ve been thinking clearly’, (6) ‘I’ve been feeling close to other people’, (7) ‘I’ve been able to make up my own mind about things’. Response options were: 1 (All the time), 2 (Frequently), 3 (Every now and then), 4 (Rarely), and 5 (Never). Response options ‘All the time’ and ‘Frequently’ represent high/good mental health in descriptive analyses. The 7-item scale is used as a continuous latent variable in structural equation modeling. In the current study, the scale showed good internal consistency with Cronbach’s α = 0.91.

Self-esteem was measured using three items measuring students’ positive conceptions of self and their conceptions of what others think about them. Students responded to the following items/claims: (1) ‘I like myself’, (2) ‘I am good enough as I am’, (3) ‘Others my age like me’ using five response categories: 1 (Strongly agree), 2 (Agree), 3 (Neither agree nor disagree), 4 (Disagree), and 5 (Strongly disagree). Response options ‘Strongly agree’ and ‘Agree’ represent high/good self-esteem in descriptive analyses. The three-item scale is used as a continuous latent variable in structural equation modeling. In the current study, the scale showed good internal consistency with Cronbach’s α = 0.89.

Gender and age were recorded by asking participants to select the appropriate alternatives from a list.

### 2.4. Statistical Analysis

Descriptive statistics were used to examine the prevalence of loneliness, mental wellbeing, and high self-esteem among all four Nordic countries. Chi-square was used to analyze the bivariate associations between gender, country, and mental health indicators. Descriptive analyses were performed with SPSS, version 23.0 (IBM Corp., Armonk, NY, USA).

Structural equation modeling was used to analyze to what extent loneliness explains the variance in mental well-being and self-esteem. Analyses were carried out using Mplus v.7.0 statistical package [37]. The parameters were estimated using the maximum likelihood robust (MLR) estimation method, which is robust to the non-normality of observed variables. Cases with missing values were included in the analyses and treated with the missing at random data procedure in Mplus. No items included in the analysis exceeded 2.7% missing values.

Structural equation modeling was performed according to the following steps. First, the structure of positive mental health measures (mental well-being and self-esteem) was determined, and two latent factors were estimated separately for all countries. Secondly, it was analyzed to what extent perceived loneliness explains the variance in mental wellbeing and self-esteem by estimating paths (regression coefficients) from perceived loneliness to the two latent factors: mental wellbeing and self-esteem. Multigroup invariance analysis was used to analyze the country differences by estimating models simultaneously for countries (configural invariance) and by adding constraints first to factor loadings (metric invariance) and second to regression coefficients.

The model fit was evaluated using the chi-square test, the root mean square error of approximation (RMSEA), the comparative fit index (CFI), the Tucker–Lewis index (TLI), and standardized root mean square residual (SRMR). As Chi-square is highly sensitive to large sample size, relative measures for the goodness of fit are recommended in addition to the chi-square test [38]. The following cut-off values were used: RMSEA < 0.06; SRMR < 0.08; CFI > 0.95; TLI > 0.95 [39]. Goodness of fit in invariance testing was also analyzed by ΔCFI, and ΔRMSEA with a criterion of change being less than –.010 in CFI and 0.015 in RMSEA [40] for factor loadings (models M1 and M2) and by scaled Chi-square test [41] for regression coefficients (models M2 and M3). The significance of country differences in regression coefficients was tested by calculating new parameters for the difference in each regression coefficient and testing the estimates of parameters against the value 0.

## 3. Results

### 3.1. Prevalence of Loneliness, Mental Wellbeing, and High Self-Esteem

The prevalence of 15-year-old adolescents with frequent loneliness, high mental well-being, and high self-esteem are presented in Table 1. In general, 14% of Nordic adolescents report feeling lonely often. The prevalence of loneliness was highest in Finland (19.2%) and Iceland (17.1%). The lowest prevalence of loneliness was in Denmark (7.7%).

Results show that in Nordic countries the majority of adolescents feel optimistic (62.2%), useful (57.7%), and relaxed (54.2%). Most adolescents also report dealing well with problems (58.3%), thinking clearly (63.3%), feeling close to other people (65.8%), and being able to make up their own mind (67.1%). Country differences in mental well-being were consistent. The highest prevalence of experiencing positive mental well-being across all seven items measuring mental wellbeing was among adolescents in Denmark. The second highest percentages were reported by Swedish adolescents, third by Icelandic adolescents, and Finnish adolescents reported the lowest prevalence for positive mental health in all items except for ‘being able to make up my own mind’ in which Icelandic adolescents reported the lowest rates. The majority of Nordic adolescents had positive evaluations of items measuring self-esteem: ‘I like myself’ (70.3%), ‘I am happy being the way I am’ (67.8%), and ‘Other people my age generally like me’ (68.6%). In line with previous results, Danish and Swedish adolescents reported the highest prevalences of high self-esteem and Finnish adolescents the lowest (Table 1).

Gender differences in mental well-being, self-esteem, and loneliness are presented in Table 2. In total, the prevalence of positive mental well-being among boys was higher than that among girls, and the association between gender and mental well-being items was significant in all except for the item “feeling close to other people”. The same pattern was seen in self-esteem and loneliness. Boys had higher self-esteem compared to girls and feelings of loneliness were more frequent among girls in all Nordic countries (Table 2).

### 3.2. Relationships between Loneliness and Positive Mental Health

The correlations, means, and standard deviations for items measuring mental wellbeing, self-esteem, and loneliness are presented in Table 3. All correlations were statistically significant (*p* < 0.001). The highest correlations were within the mental well-being scale between the items ‘feeling optimistic about future’ and ‘feeling useful’ (*r* = 0.72) and between ‘dealing well with problems’ and ‘thinking clearly’ (*r* = 0.72). Within items measuring self-esteem, the highest correlation was between ‘I like myself’ and ‘I am good enough as I am’ (*r* = 0.85). Loneliness had the strongest negative correlations with ‘Feeling useful’, ‘I like myself’, and ‘I am good enough as I am’ (all *rs* = −0.42).

**Figure 1:**
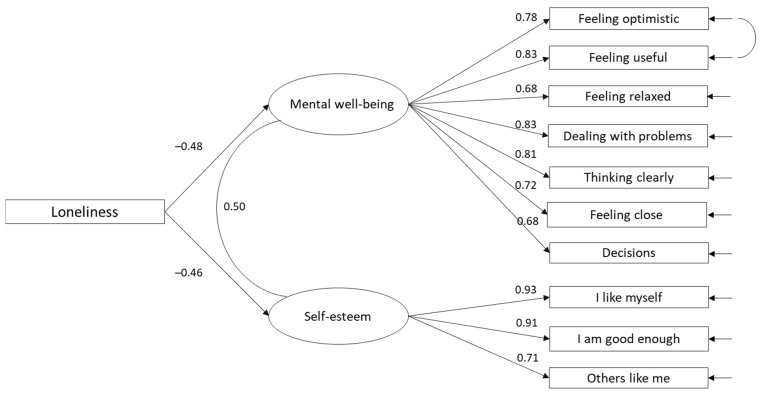
Perceived loneliness as a predictor for low mental well-being and low self-esteem (*n* = 5751). Standardized beta coefficient reported. All estimated parameters were statistically significant at level *p* < 0.001.

To answer our research question, to what extent does loneliness explains variance in positive mental well-being and self-esteem, we estimated the regression coefficients from loneliness to two latent factors: mental well-being (‘feeling optimistic’, ‘feeling useful’, ‘feeling relaxed’, ‘dealing well with problems’, ‘thinking clearly’, ‘feeling close’, ‘being able to make up my own mind’) and self-esteem (I like myself, I am good enough, others like me). The covariance between ‘feeling optimistic’ and ‘feeling useful’ was also estimated in the final model based on high modification indices (Figure 1). The fit indices showed good fit for the model in which the regression coefficients were added from loneliness to mental wellbeing and self-esteem: χ²(41) = 510.80, *p* < 0.001; CFI = 0.98, TLI = 0.97, RMSEA = 0.05, SRMR = 0.03. All items measuring mental wellbeing and self-esteem had high factor loadings varying from 0.69 to 0.93. The regression coefficient between loneliness and mental well-being was −0.48 (*p* < 0.001), and between loneliness and self-esteem −0.46 (*p* < 0.001), indicating that loneliness is a significant risk factor for mental well-being and self-esteem among adolescents in Nordic countries. Loneliness explained 23% of the variance in mental wellbeing and 21% of the variance in self-esteem. All estimated parameters were statistically significant at level *p* < 0.001 and are presented in Figure 1.

### 3.3. Country-Level Differences in Loneliness and Mental Health

**Figure 2:**
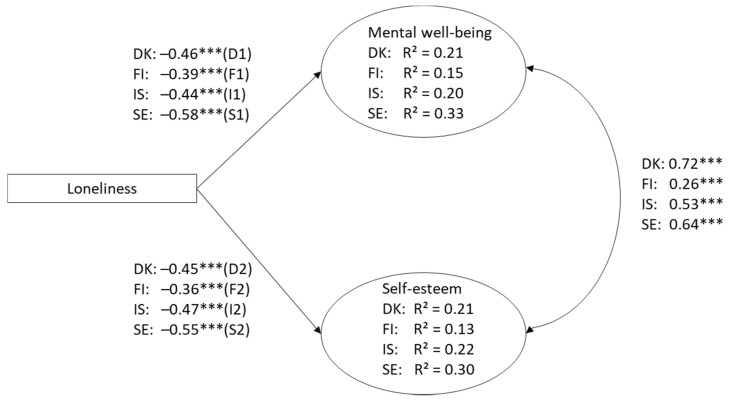
Regression coefficients of loneliness predicting/explaining low mental well-being and low self-esteem in subgroups formed by country (Denmark, DK; Finland, FI, Iceland, IS; Sweden, SE). Factor loadings set equal, standardized beta coefficient estimates reported. *** *p* < 0.001.

Multi-group comparisons were used to analyze how strongly loneliness is associated with mental well-being and self-esteem in Denmark, Finland, Iceland, and Sweden. Using a multi-group model, we estimated the model simultaneously for all countries. First with no constraints in factor loadings (model M1) and secondly by setting similar factor loadings (model M2). Nested models were compared by ΔCFI = −0.008 and ΔRMSEA = 0.002, indicating that factor loadings were the same across four countries. In model M2 (Figure 2) the factor loadings were set equal for all countries and the interest was in regression coefficients. The fit indices showed good fit for the model estimated simultaneously for all countries: χ²(188) = 1242,01; *p* < 0.001; CFI = 0.96; TLI = 0.95; RMSEA = 0.06, SRMR = 0.06. Loneliness was a significant risk factor for both mental wellbeing and self-esteem in all four Nordic countries. Based on regression coefficients, loneliness explained the highest variance in adolescents’ mental health among Swedish adolescents (mental wellbeing *R*^2^ = 0.33; self-esteem *R*^2^ = 0.30), and the second highest among Icelandic adolescents (mental wellbeing *R*^2^ = 0.20; self-esteem *R*^2^ = 0.22) and Danish adolescents (mental wellbeing *R*^2^ = 0.20; self-esteem *R*^2^ = 0.21) and least among Finnish youth (mental wellbeing *R*^2^ = 0.15; self-esteem *R*^2^ = 0.13). All estimated parameters were statistically significant and are presented in Figure 2.

Finally, we analyzed if loneliness was an equally strong predictor of mental wellbeing and self-esteem in Denmark, Finland, Iceland, and Sweden. To do this, we estimated a model in which regression coefficients were set equal between groups (model M3) and compared the model fit of the constrained model with the model with freely estimated regression coefficients (model M2) using a scaled chi-square test (SB χ²(6) = 52.44, *p* < 0.001). The scaled chi-square test indicated that regression coefficients cannot be set equal between different countries (SB χ²(6) = 52.44, *p* < 0.001), meaning that the strength in which loneliness predicts mental health varies between Nordic countries (Table 4).

Finally, pairwise comparisons in regression coefficient differences were analyzed by calculating new parameters for each country difference (e.g., T1 = D1–F1). Results of the pairwise comparisons show that the association (regression coefficient) between loneliness and mental wellbeing is significantly stronger/higher in Sweden compared to that in Denmark (D1–S1 = −0.171, *p* < 0.001), Finland (F1–S1 = −0.188, *p* < 0.001), and Iceland (I1–S1 = −0.161, *p* < 0.001). The pairwise comparisons in loneliness and self-esteem show that the regression coefficient is significantly lower in Finland compared to that in Denmark (D2–F2 = −0.181, *p* = 0.003), Iceland (F2–I2 = 0.119, *p* = 0.009), and Sweden (F2–S2 = 0.261, *p* < 0.001). In addition, the regression coefficient between loneliness and self-esteem is higher in Sweden compared to that in Iceland (I2–S2 = 0.142, *p* = 0.001; please see parameters and regression coefficients in Figure 2).

## 4. Discussion

Using cross-national data collected from among 15-year-old adolescents in Denmark, Finland, Iceland, and Sweden, we found that the majority of Nordic adolescents are doing well in terms of mental wellbeing and self-esteem, but for nearly 15%, feelings of loneliness were frequent, and loneliness was a significant risk factor for participants’ mental well-being and self-esteem in all four countries.

Feelings of loneliness were most frequent in Finland with 19% reporting loneliness and the lowest prevalence was in Denmark (8%). Girls reported higher rates of loneliness compared to boys in all four Nordic countries. Previous research shows that boys generally report higher rates of loneliness when loneliness is measured indirectly while girls report higher rates when loneliness is measured with one direct question, the global feeling of loneliness [42]. Thus, our results are consistent with previous findings. In addition, social stigma related to loneliness [43] may explain the differences between direct and indirect measures of loneliness.

More than half of the participants agreed with the positive mental health statements (e.g., Feeling optimistic about the future, 62%; Feeling relaxed, 54%) and over two-thirds agreed with the positive self-esteem statements (e.g., I like myself, 71%; I am good enough as I am, 68%). This is a positive result as we know that adolescents’ positive experience of mental health is essential for future development [20] and the development of the abilities to cope with normal stressors of daily life [16] (p. 12). These results are similar to the findings in a study of US adolescents showing that the majority feel happy, interested in life, and satisfied almost every day or daily [22] and with the results of a Chinese study where over half the participants were classified as having high mental well-being [21].

Country differences were evident, with Denmark and Sweden reporting systematically higher rates in mental well-being measures and self-esteem and Finnish adolescents the lowest. This is in line with the results from a recent Nordic comparison of excellent self-rated health where Swedish and Danish adolescents reported higher rates than did Icelandic and Finnish adolescents [29]. In the present study, boys reported higher rates of mental well-being for all items on the mental well-being scale than did girls in all four countries. This finding supports earlier results where boys scored higher on a mental well-being scale compared with girls [44].

The results of the present study suggest a strong relationship between loneliness and mental health among Nordic adolescents. Within each country there was a similar pattern where loneliness was found to be a determinant of lower mental wellbeing and self-esteem. The strength of association between loneliness and mental health indicators (mental well-being and self-esteem) was strongest in Sweden with loneliness explaining 33% of the variation in mental well-being and 30% of the variation in self-esteem. The association between loneliness and low self-esteem has been explained as a diminishing effect from loneliness to self-esteem, particularly for those experiencing long-term and chronic loneliness. Low self-esteem may be a direct reflection of the experience of loneliness and an individuals’ dissatisfaction with their social life may be linked to broader dissatisfaction resulting in lower self-esteem [27].

The present study had several limitations. The data were cross-sectional and thus limit causal inference from the findings regarding associations between variables. However, the associations between loneliness and mental well-being, and loneliness and self-esteem indicate that loneliness is a potential risk factor for worse/lower mental health during adolescence. This model can be tested in future longitudinal studies examining potential causal pathways. There are other factors in addition to loneliness related to mental health that were not included in the present study. In the present study, loneliness was measured using a direct single-item measure. Although research has shown that one direct question on loneliness is strongly correlated with the multi-item UCLA (University of California, Los Angeles) Loneliness Scale measuring the concept indirectly [8], participants in the present study may have had different conceptions of loneliness and the dimensionality of loneliness could not be evaluated. The findings reported rely on self-reports rather than interviews or assessments by others (e.g., teachers and parents); however, this is likely the best method to gather data when it comes to large population-based surveys with adequate reliability and validity among adolescents [30,45]. Data used in this study were collected from four Nordic countries that are quite homogenous in social, cultural, and geographical aspects [29]. While results are comparable across the four Nordic nations, future cross-national research could consider the extent to which the findings of the present study are applicable outside of the Nordic countries.

Research suggests that children and adolescents have a fundamental understanding of what loneliness is and that loneliness can be reliably measured in these age groups [46]. However, despite the ease of single-item questions, such measures require an individual to identify loneliness and label himself or herself as “lonely” and, therefore, they carry an element of social stigma [47]. This could potentially lead to an underestimation of the prevalence of loneliness.

## 5. Conclusions

Although the majority of adolescents appear to be doing well in terms of self-rated mental well-being and self-esteem, about 30 to 40% of adolescents experience mental well-being less than frequently and do not agree with positive self-esteem statements, and 15% feel frequent loneliness. There is a need to further investigate both factors that improve mental health and barriers to positive mental health in adolescents and to broaden the scope of research to understand the country-level factors associated with the country differences. The finding that loneliness is strongly associated with lower mental well-being and self-esteem demonstrates the importance of targeting interventions not only to mental health promotion but also to factors associated negatively with mental health. A recent meta-analysis showed that interventions could reduce loneliness among youth (a moderate effect size) [47], which implies that actions taken to reduce loneliness may be an effective way of supporting adolescents’ mental health.

## Figures and Tables

**Table 1:** Prevalence of loneliness, positive mental health, and high self-esteem in Denmark, Finland, Iceland, and Sweden (*n* = 5717 to 5861).

Measure	All	Denmark	Finland	Iceland	Sweden	*p* ^d^
Loneliness ^c^						
Feeling lonely	14.3	7.7	19.2	17.1	11.9	<0.001
Mental well-being ^a^						
Feeling optimistic about the future	62.4	76.7	46.4	59.4	69.2	<0.001
Feeling useful	57.9	67.4	47.8	55.7	62.0	<0.001
Feeling relaxed	54.3	61.5	48.0	49.5	60.9	<0.001
Dealing with problems well	58.4	71.2	46.0	53.9	65.7	<0.001
Thinking clearly	63.4	74.4	55.8	59.8	67.1	<0.001
Feeling close to other people	65.9	75.6	59.1	61.9	70.5	<0.001
Able to make up my own mind	67.3	88.4	68.5	48.6	80.6	<0.001
Self-esteem ^b^						
I like myself	70.5	72.4	66.8	70.0	72.0	0.001
I am good enough as I am	67.8	71.5	66.2	66.0	69.3	0.003
Others my age like me	68.7	72.5	60.4	68.0	72.7	<0.001

^a^ Mental well-being (The Short Warwick-Edinburgh Mental Well-being Scale, SWEMWBS): percentages for response options ‘all the time’ and ‘frequently’. ^b^ Self-esteem: percentages for response options ‘strongly agree’ and ‘agree’ ^c^ Loneliness: percentages for response options ‘quite often’ and ‘very often’ (Denmark, DK; Finland, FI; Iceland, IS) and response options ‘always’ and ‘most of the time’ (Sweden, SE ^d^ Chi-square for association between mental health indicators (mental well-being, self-esteem, loneliness) and country.

**Table 2:** Prevalences of adolescent boys (*n* = 2740 to 2840) and girls (*n* = 2883 to 2922) reporting loneliness, positive mental health, and high self-esteem.

Measure	All			Denmark			Finland			Iceland			Sweden		
	Boys	Girls	*p*	Boys	Girls	*p*	Boys	Girls	*p*	Boys	Girls	*p*	Boys	Girls	*p*
Loneliness ^c^															
Feeling lonely	10.7	17.9	***	5.6	9.9	***	12.5	25.5	***	13.7	19.6	***	8.3	14.8	***
Mental well-being ^a^															
Feeling optimistic about the future	67.4	57.7	***	85.5	68.4	***	47.5	45.3		62.9	56.7	**	78.1	61.5	***
Feeling useful	64.8	51.3	***	76.1	59.0	***	52.9	42.7	**	61.9	50.4	***	71.2	54.1	***
Feeling relaxed	63.8	45.2	***	73.0	50.7	***	57.3	38.9	***	55.8	43.7	***	74.9	48.6	***
Dealing with problems well	65.6	51.7	***	77.4	65.4	**	53.8	38.2	***	60.2	48.6	***	75.1	57.4	***
Thinking clearly	69.6	57.5	***	82.0	67.3	***	60.9	50.4	**	65.0	55.7	***	75.7	59.4	***
Feeling close to other people	67.4	64.5		78.0	73.2		53.7	64.2	**	62.8	61.8		78.0	64.0	***
Able to make up my own mind	73.0	61.8	***	89.5	87.3		68.4	68.5		58.6	39.5	***	87.9	74.0	***
Self-esteem ^b^															
I like myself	81.0	60.5	***	84.9	60.6	***	77.3	56.5	***	79.4	61.7	***	83.7	61.5	***
I am good enough as I am	76.5	59.6	***	82.0	61.5	***	75.5	57.1	***	72.7	59.8	***	79.5	60.1	***
Others my age like me	74.5	63.1	***	79.3	66.0	***	68.3	52.6	***	72.6	64.0	***	79.1	67.3	***

^a^ Mental well-being (SWEMWBS): percentages for response options ‘all the time’ and ‘frequently’ ^b^ Self-esteem: percentages for response options ‘strongly agree’ and ‘agree’ ^c^ Loneliness: percentages for response options ‘quite often’ and ‘very often’ (DK, FI, IS) and response options ‘always’ and ‘most of the time’ (SE) *p*-value: Chi-square for association between mental health indicators (mental well-being, self-esteem, loneliness) and gender; *p* < 0.001 ***; *p* < 0.01 **.

**Table 3:** Pearson correlations between mental health indicators (mental wellbeing: items 1–7; self-esteem: items 8–10; loneliness: item 11) applied in the present study.

Item	1	2	3	4	5	6	7	8	9	10	11
1. Loneliness	−										
2. Feeling optimistic about the future	−0.36 *	-									
3. Feeling useful	−0.42 *	0.72 *	−								
4. Feeling relaxed	−0.32 *	0.50 *	0.55 *	−							
5. Dealing with problems well	−0.39 *	0.61 *	0.66 *	0.59 *	−						
6. Thinking clearly	−0.37 *	0.61 *	0.63 *	0.56 *	0.72 *	−					
7. Feeling close to other people	−0.33 *	0.55 *	0.59 *	0.48 *	0.57 *	0.59 *	−				
8. Able to make up my own mind	−0.33 *	0.51 *	0.54 *	0.46 *	0.57 *	0.58 *	0.51 *	−			
9. I like myself	−0.42 *	0.47 *	0.53 *	0.37 *	0.44 *	0.43 *	0.39 *	0.36 *	−		
10. I am good enough as I am	−0.42 *	0.43 *	0.52 *	0.36 *	0.43 *	0.42 *	0.38 *	0.35 *	0.85 *	−	
11. Others my age like me	−0.41 *	0.39 *	0.46 *	0.31 *	0.39 *	0.36 *	0.41 *	0.32 *	0.66 *	0.64 *	−
*M*	3.29	2.33	2.41	2.49	2.40	2.30	2.24	2.19	2.13	2.18	2.17
*SD*	0.85	1.02	0.98	0.99	0.96	0.96	0.99	1.03	1.05	1.08	0.95
*N*	5861	5753	5727	5740	5737	5723	5726	5725	5767	5753	5717

* *p* < 0.01.

**Table 4:** Model fit indices for Denmark, Finland, Iceland, and Sweden and multi-group invariance across Nordic countries.

Model	χ^2^(*df*)	*p*	CFI	TLI	RMSEA	SRMR
Separate models						
Denmark	χ^2^(41) = 240.38	<0.001	0.95	0.93	0.08	0.05
Finland	χ^2^(41) = 194.89	<0.001	0.97	0.96	0.06	0.03
Iceland	χ^2^(41) = 218.45	<0.001	0.98	0.97	0.04	0.03
Sweden	χ^2^(41) = 353.54	<0.001	0.95	0.94	0.07	0.05
Country invariance						
M1	χ^2^(164) = 1005.38	<0.001	0.97	0.96	0.06	0.04
M2	χ^2^(188) = 1242.01	<0.001	0.96	0.95	0.06	0.06
M3	χ^2^(194) = 1294.31	<0.001	0.96	0.95	0.06	0.07

Fit Indices: CFI, comparative fit index; TLI, Tucker Lewis index; RMSEA, root mean square error of approximation; SRMR, standardized root mean square residual.

## Data Availability

The data presented in this study are available on reasonable request from the corresponding author or HBSC Data Management Centre, University of Bergen, Norway (dmc@hbsc.org).
